# Longnon-coding RNA BLACAT2 promotes gastric cancer progression via the miR-193b-5p/METTL3 pathway

**DOI:** 10.7150/jca.50403

**Published:** 2021-04-02

**Authors:** Hao Hu, Qi Kong, Xiao-xu Huang, Hao-ran Zhang, Kai-feng Hu, Yan Jing, Yang-fan Jiang, Yue Peng, Long-chao Wu, Qi-sheng Fu, Li Xu, Ya-bin Xia

**Affiliations:** 1 Department of Gastrointestinal Surgery, The First Affiliated Hospital of Wannan Medical College, Wuhu, AnHui, 241001, P.R. China.; 2 Wannan Medical College, Wuhu, AnHui, 241001, P.R. China.

**Keywords:** gastric cancer, lncRNA-BLACAT2 (BLACAT2), miR-193b-5p, METTL3, proliferation, apoptosis

## Abstract

Gastric cancer is one of the leading prevalent and malignant cancers worldwide, especially in east Asia. However, the in-depth molecular mechanism underlying gastric cancer progression remains uncertain. Recently, studies have identified that long non-coding RNA (lncRNA) could play critical roles in the tumorigenesis of multiple types of cancer. Studies on long non-coding RNA BLACAT2 have proven that it participates in bladder cancer and colorectal cancer regulation and was identified as highly expressed using the cBioPortal for Cancer Genomics in gastric cancer. However, the precise function of lncRNA-BLACAT2 in the carcinogenesis and progression of gastric cancer remains largely unexplored. Our study discovered that lncRNA-BLACAT2 was significantly upregulated in gastric cancer. Different studies have illustrated that BLACAT2 promoted gastric cancer progression through regulating proliferation, migration, invasion, and apoptosis in terms of biological function.

Furthermore, BLACAT2 was verified to perform its function through interaction with miR-193b-5p using a luciferase reporter assay. On the other hand, MiR-193b-5p specific inhibitor treatment reversed the inhibitory effect of BLACAT2 on cell biological functions. Additional studies also discovered that Methyltransferase Like 3 (METTL3) was the downstream target of miR-193b-5p. Subsequently, restoration of METTL3 eliminated the suppressive effect of proliferation or the promotive effect of apoptosis caused by BLACAT2 knockdown. To sum up, these experimental results demonstrated that BLACAT2 acted as an oncogene in gastric cancer progression through the regulation of the miR-193b-5p/METTL3 pathway, hence providing new insights regarding the pathogenesis of gastric cancer.

## Introduction

Gastric cancer is the second leading cause of cancer-related death and the fifth most commonly diagnosed malignancy in the world 1, 2. More seriously, its morbidity is gradually growing yearly. Studies have shown that most of these patients are diagnosed at the advanced stage, which is ascribed to the fact that there are no specific symptoms at the very early stage 3. Moreover, other studies have demonstrated that the existing therapeutic strategies, such as surgical operation, radiotherapy, and chemotherapy, are not satisfactory because of the aggressive proliferation, extensive invasion, and lymphatic metastasis of gastric cancer 4, 5. Still, the molecular mechanism for the malignant development of gastric cancer remains unknown. Therefore, it is imperative to investigate gastric cancer development mechanisms and seek potential novel therapeutic biomarkers 6.

Recently, long non-coding RNAs (lncRNAs) have been confirmed as novel members of the non-coding RNA family, which are transcribed RNA molecules with a minimum length of 200 nucleotides without any protein-coding capability 7. They modulate gene expression at either the transcriptional or post-transcriptional level 8. Furthermore, research has confirmed their pivot functions in diverse biological processes, including alternative splicing, nuclear import, cell differentiation, and sponging microRNA 9. Besides, they have been reported to be involved in various biological functions, such as proliferation and apoptosis, metastasis, cellular metabolism, and cancer progression 10. In the present study, we identified lncRNA-00958 as a potential diagnostic marker involved in various tumors, and we named it bladder cancer-associated transcript 2 (BLACAT2). Previous studies have revealed that it is associated with cancer progression and contributes to cancer treatment and prognosis. Elsewhere, it is reported that it promotes lymph angiogenesis and lymphatic metastasis in bladder cancer 11.

Moreover, other studies have proved that it acts as an oncogene and contributes to disease progression, chemoresistance, and poor patient survival with colorectal cancer 12. After database analysis in gastric cancer, this transcript was observed to be up-regulated 13. However, its exact biological role in gastric cancer is yet to be completely elucidated.

Taken together, we used gene microarray screening and RT-PCR and found that lncRNA-BLACAT2 is significantly upregulated in gastric cancer tissues compared to the corresponding normal tissues. Besides, for the first time, our study has revealed that this transcript regulates proliferation, apoptosis, migration, and invasion through the miR-193b-5p/METTL3 pathway.

## Material and methods

### *In silico* analysis using the ONCOMINE database

To investigate the expression pattern of METTL3 in gastric cancer, we used ONCOMINE dataset (www.oncomine.com). Data was collected and analysed using R 3.0.2 software.

### Bioinformatics prediction

The web‐based online tool TargetScanHuman (http:// www.targetscan.org/) was used for targeting between lncRNA and miRNA prediction. The online softwares (Target Scan, miRbase, miRwalk, PicTar) were used for prediction of the interaction between miR‐193b-5p and the 3′‐untranslated region (3′‐UTR) of METTL3.

### Sample collection

Primary gastric cancer tissues and the normal samples were acquired from the gastric cancer patients who underwent surgical resection at the department of gastrointestinal surgery, the first affiliated hospital of Wannan Medical College. These tissues were immediately snap‐frozen in the liquid nitrogen before further analysis. Our study was approved by the Research Ethics Committee of Wannan Medical College and all samples were collected with participants' written informed consent.

### Cell culture

Gastric cancer cell lines (AGS, MGC803, HGC27, MKN45, BGC823, SGC7901, MKN28) and the immortalized human gastric mucosal cell line GES-1were obtained from the Cell Bank of the Chinese Academy of Sciences (Shanghai, China). The immortalized human gastric mucosal cell line GES-1 obtained from the Cell Bank of the Chinese Academy of Sciences (Shanghai, China). Cells were routinely cultured with RPMI-1640 medium supplemented with 10% fetal bovine serum and 1% antibiotics (100 μg/mL streptomycin and 100 units/mL penicillin) at 37 °C in an incubator with a 5% CO2 atmosphere. The relative humidity was 90%. Medium was replaced every 3 days.

### Cell transfection

Short hairpin RNAs (shRNA) for lncRNA BLACAT2 or METTL3 (sh‐BLACAT2 or sh‐METTL3), miR‐193b-5p mimics, miR‐193b-5p inhibitor, METTL3 oe and their relative negative controls (sh‐Nc, Nc mimics, Nc inhibitor) were transfected into AGS or MGC803 cells using Lipofectamine 2000 (Invitrogen, Carlsbad, CA). All transfection plasmids were purchased from GenePharma Corporation (Suzhou, Jiangsu, China). Then, the cells were collected 48 hours after transfection.

### Quantitative reverse-transcription polymerase chain reaction (qRT-PCR)

Total RNAs were isolated from tumor cells using TRIzol (Invitrogen, Grand Island, NY). A complementary DNA (cDNA) synthesis kit (Aidlab, Beijing, China) was then used to initiate cDNA synthesis. Quantitative reverse transcription‐polymerase chain reaction (qRT‐PCR) was carried out on an ABI 7900HT sequence detection machine (Thermo Fisher Scientific, Waltham, MA) with SYBR Green qPCR Mix (Aidlab). All experiments were done in duplicate. The 2-ΔΔCt method was utilized to compute the results. (METTL3 Forward Primer: TTGTCTCCAACCTTCCGTAGT, Reverse Primer: CCAGATCAGAGAGGTGGTGTAG; BLACAT2 Forward Primer: GCAGAATCGCTTGAACCCAGGAG, Reverse Primer: AGATGGAGTTTCGCTCTTGTTGCC; mir-193b-5p Forward Primer: CGGGCCGGGGTTTTGAGGGC, Reverse Primer: CAGCCACAAAAGAGCACAAT).

### Western blotting

Total cellular proteins were extracted using a total protein extraction kit (Beyotime, China). Cell lysates were separated by 10% SDS-PAGE gel electrophoresis and transferred to a nitrocellulose membrane. The membranes were blocked with 5% nonfat milk and incubated with the primary antibodies and then incubated with species-specific secondary antibodies. The following antibodies were used at the indicated concentrations METTL3 (1:1000, Abcam, USA), GAPDH (1:1000, CST, USA).

### Cell viability assay (CCK8 assay)

AGS and MGC803 cells were prepared and seeded into 96-well plates at a density of 2000-3000 cells per well with 100μL medium containing 10% serum. Each group contained 5 wells. Cell Counting Kit-8 (CCK-8, Dojindo, Japan) solution (10 μL) was added to each well at 0, 24, 48, 72 and 96h, respectively. Cell viability was measured using a microplate reader (BIO-TEK) at the absorbance of 450 nm. This experiment was repeated twice at each time point.

### Cell apoptosis analysis

In the apoptosis assay, cells were stained with propidium iodide and Annexin V-fluorescein isothiocyanate (BD Pharmingen) in accordance with the manufacturer's instructions. Briefly, cells were washed with PBS and resuspended in 1× Binding Buffer, then, 5 μl FITC Annexin V and 5 μl PI were added to 100 μl of the cell suspension and incubated for 15 min in the dark. After incubation, 400 μl 1× Binding Buffer was added. Apoptosis was analyzed by FACS using the Cell-Quest software. Annexin V-FITC-positive and PI-negative cells were regarded as apoptotic.

### Wound healing assay

AGS and MGC803 Cells were seeded into 6-well plates and then incubated for 24 h until they were 80-90% confluent. Wounds were created on these cells with a sterile pipette tip and cells were cultured in the medium without FBS. After wounding (time 0 h and 24 h), photographs were taken to assess the capacity of the cells to migrate into the wound area (original magnification, 100 ×). Experiments were performed in triplicate and repeated three times.

### Transwell invasion assay

We carried out invasion assays by adding 100μL matrigel (BD Bioscience, Franklin Lakes, NJ) into top chamber of transwell and placed 40000 cells onto the matrigel. 24 h later, the transwell invasion assay was terminated and we proceeded with staining with 0.1% crystal violet. Then cells were counted under digital microscopy.

### Plate colony formation assay

Plate colony formation assay was carried out using six-well plates. Cells (1 × 103) were seeded in each well with 2ml 1640 supplemented with 10% FBS. The culture media were refreshed once 3 days. After 14 days, the resulting colonies were fixed with methanol at -20 °C for 5 min, and then stained with crystal violet. Only clearly visible colonies (diameter > 50 μm) were calculated.

### *In vivo* tumor xenograft model

Six-week-old male nude (nu/nu) mice (SLAC, Shanghai, China) were injected subcutaneously in the right flank with the stable single cell clones of AGS cells (2 × 107 ) with or without BLACAT2 silencing in 100 μl serum-free RPMI 1640 medium. After 6 weeks, mice were sacrificed, and their tumors were dissected, Vernier calipers were used for tumor sizes (volume = 1/2 × length × width2 ) measurement.

### Assay of dual luciferase reporter

Reporter plasmids were constructed by adding wild/mutation BLACAT2 or METTL3 3′‐UTR sequence to the pGL3 vector (Promega, Madison, WI). Lipofectamine 2000 was then used and miR‐193b-5p mimics combined with reporter plasmids were cotransfected into 239T cells. We used the Dual‐Luciferase Reporter Assay System (Promega, Sunnyvale, CA) to detect firefly and Renilla luciferase activities after culturing for 48 hours according to the manufacturer's instructions.

### Statistical analysis

All results were from three independent experiments, analyzed by SPSS version 21.0 (IBM Corporation) and GraphPad Prism5 (San Diego, CA) software, expressed as the mean ± SD. All P-values were determined from 2-tailed tests and differences with a P-value < 0.05 was considered to be statistically significant.

## Results

### The expression of lncRNA-BLACAT2 was significantly upregulated in gastric cancer tissues and cell lines

**Figure 1:**
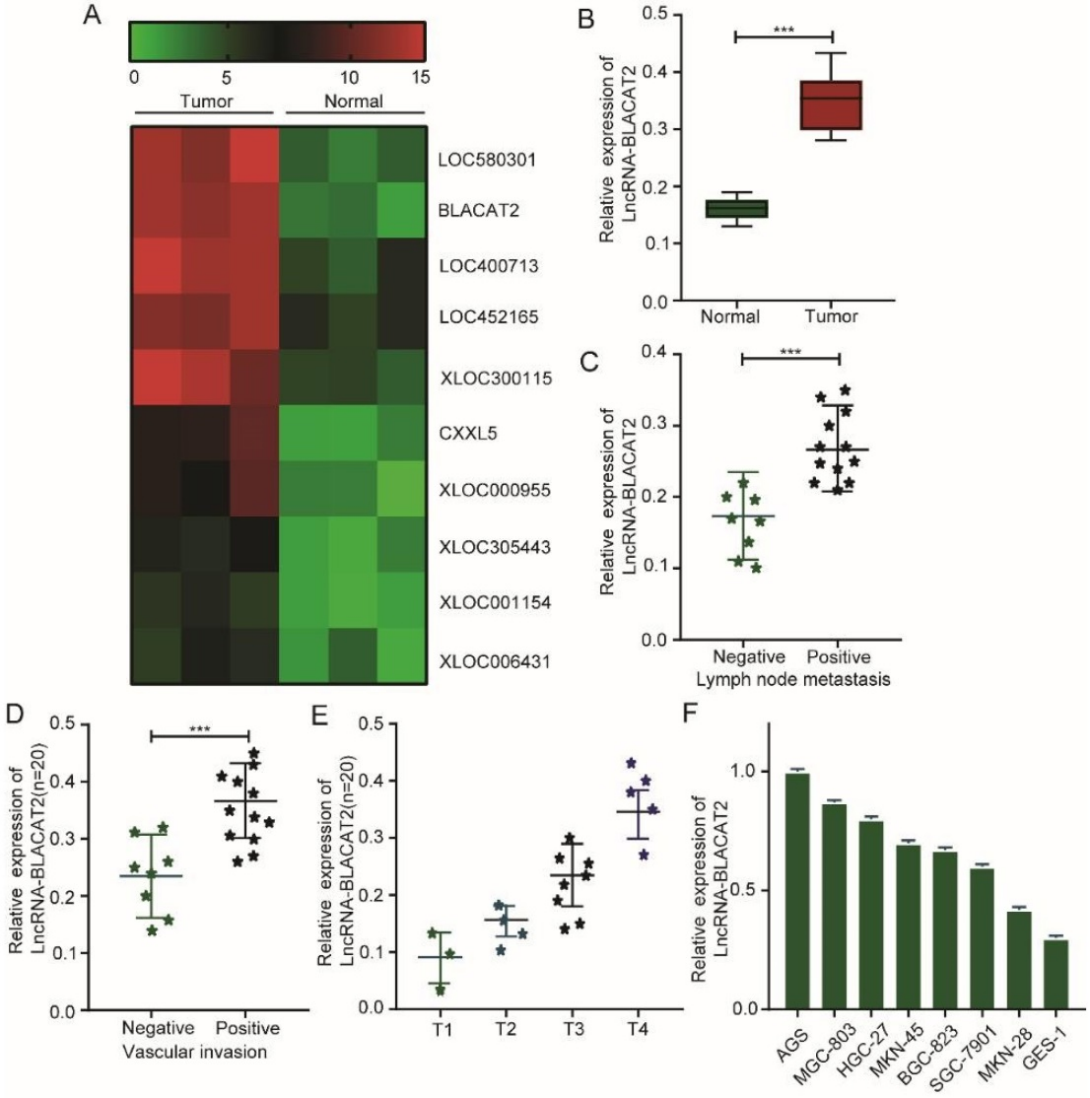
** Increased BLACAT2 expression in gastric cancer tissues and cell lines. A:** Whole-genome expression profile showing remarkable up-regulation of BLACAT2 in gastric cancer cell tissues compared to normal tissues. **B:** qRT‐PCR was used to analyze BLACAT2 expression in gastric cancer tissues and 20 paired normal tissues. **C:** The expression of BLACAT2 was higher in those with lymph node metastasis than those without lymph node metastasis. **D:** The expression of BLACAT2 was higher in those with vascular invasion than those without vascular invasion. **E:** The amount of BALCAT2 expression increased with the increase of T stage in gastric cancer tissues. **F:** BLACAT2 expression was upregulated in all 7 GC cell lines compared with the nonmalignant GES-1 cells. BLACAT2: lncRNA-BLACAT2; qRT‐PCR: quantitative reverse transcription-polymerase chain reaction; *p < 0.05, **p < 0.01, and ***p < 0.001. The data were denoted by mean ± SD.

Here, we screened differentially expressed lncRNAs in gastric cancer, and a genome-wide expression profiling chip was fabricated. As illustrated in **Figure 1A**, we used hotmap analysis and established that several lncRNAs, including lncRNA-BLACAT2, were substantially up-regulated in gastric cancer tissues compared to normal tissues. Subsequently, through this phenomenon, we speculated that BLACAT2 could regulate gastric cancer development. Next, we collected a total of 20 gastric cancer tissues and their paired normal controls, from which we confirmed that the BLACAT2 expression level was remarkably increased in the tumor tissues **(Figure 1B).** Then, we divided these gastric cancer tissues into two distinct groups according to either lymph node metastasis or vascular invasion condition. Here, as demonstrated in **Figure 1C-D**, we discovered that the patients with lymph node metastasis or vascular invasion displayed a relatively higher BLACAT2 expression. Besides, the amount of BALCAT2 expression increased with the increase of T stage in gastric cancer tissues **(Figure 1E)**.

Consequently, we further detected the expression of BLACAT2 in 7 gastric cancer cell lines (AGS, MGC803, HGC27, MKN45, BGC823, SGC7901, and MKN28) and one immortalized gastric mucosa cell line (GES-1). Lastly, as illustrated in **Figure 1F**, this study established that the expression of BLACAT2 was also elevated in all the cancer cell lines compared to standard cell lines. Therefore, these results suggested that this transcript could act as a potential biomarker in gastric cancer prognosis.

### Silencing of BLACAT2 inhibited gastric cancer cell proliferation, enhanced apoptosis *in vitro,* and suppressed tumor growth *in vivo*

**Figure 2:**
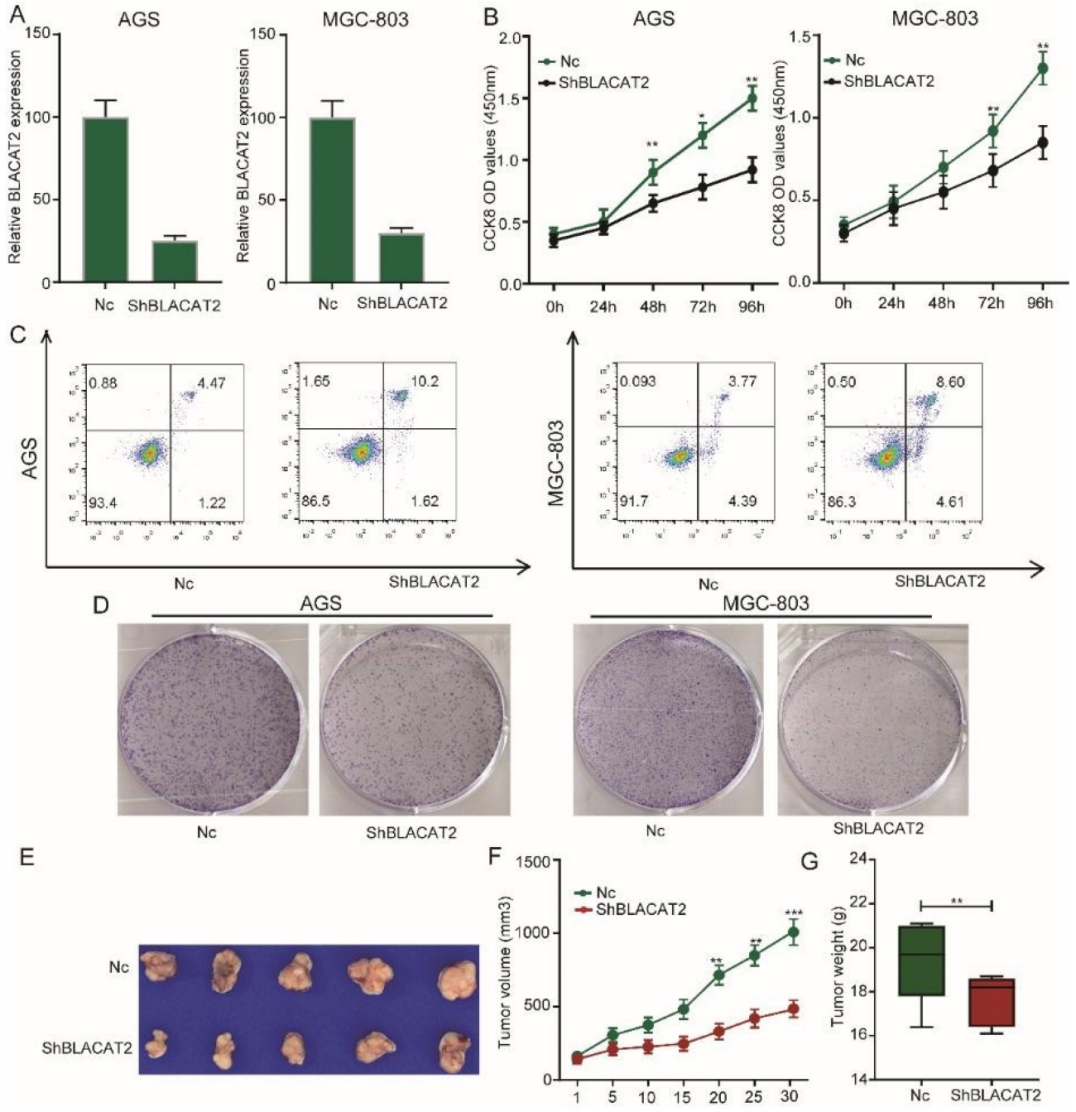
** Silencing of BLACAT2 inhibited gastric cancer cell proliferation, enhanced apoptosis *in vitro*, and suppressed tumor growth *in vivo*. A:** RT-PCR confirmed BLACAT2 knockdown efficiency in AGS and MGC-803 cells. **B:** The cell proliferation of Nc and sh-BLACAT2 in AGS and MGC-803 cells were evaluated by CCK8 assay at 0, 24, 48, 72, 96 h. The results showed that knockdown of BLACAT2 significantly inhibited the proliferation of AGS and MGC-803 cells *in vitro*. **C:** Downregulation of BLACAT2 significantly increased apoptosis in AGS and MGC-803 cells. **D:** Plate colony formation assay of AGS/BLACAT2-sh and MGC-803/BLACAT2-sh and Nc cells on regular culture plates after 14 days of culture. Relative colony numbers of BLACAT2-sh cells were significantly lower than that of Nc cells. **E:** Photographs of tumors from mice inoculated with AGS/Nc and AGS/shBLACAT2 cells. **F:** Summary of tumor volumes in mice that were measured weekly. **G:** Tumor weights of Nc and shBLACAT2 groups from E, n = 5. CCK8: Cell Counting Kit‐8; *p < 0.05, **p < 0.01, and ***p < 0.001. The data were denoted by mean ± SD.

Afterward, we sought to explore the biological function of BLACAT2 in gastric cancer. Here, we selected two gastric cancer cell lines with relatively higher BLACAT2 expression (MGC803and AGS cells) for loss-of-function experiments. Small hairpin RNA (shRNA) targeting BLACAT2, designated as sh-BLACAT2 and a mock vector marked as Nc, were transfected into AGS and MGC803 cell lines. Next, we detected the silencing effect of the shRNA in the two cell lines through RT-PCR **(Figure 2A).** Importantly, as illustrated in **Figure 2B**, the CCK8 assay demonstrated that the BLACAT2 knockdown potently inhibited the viability of gastric cancer cell proliferation. Then, we assessed the contribution of apoptosis in BLACAT2 silenced cells through flow cytometry with Annexin V and PI double staining. The results showed that both AGS and MGC803 cells transfected with sh-BLACAT2 acquired a much higher apoptosis ratio than cells transfected with Nc **(Figure 2C).** The statistical results were conducted, as shown in **Supplementary Figure 1A.** A colony formation experiment was also performed to confirm the influence of BLACAT2 on gastric cancer cells. The suppressing effects of proliferation were verified in both AGS and MGC803 cell lines **(Figure 2D)**; the statistical results were shown in **Supplementary Figure 1B.** Subsequently, we performed the xenograft experiment to detect the effects of BLACAT2 toward subcutaneous tumorigenesis *in vivo*. As shown in **Figure 2E-G,** stable silencing of BLACAT2 in AGS cells significantly inhibited tumor volume and weight *in vivo*, suggesting that this cancer-associated transcript regulated gastric cancer growth *in vivo*.

### Silencing of BLACAT2 suppressed gastric cancer cell migration and invasion *in vitro*

**Figure 3:**
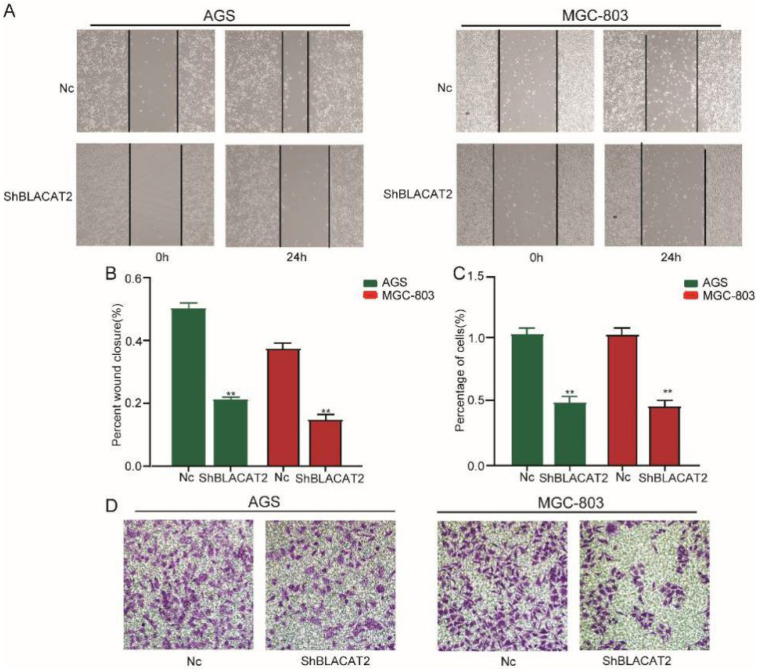
** Silencing of BLACAT2 suppressed gastric cancer cell migration and invasion *in vitro*. A:** Transwell assays in the BLACAT2-silencing group compared with the Nc group in AGS and MGC-803 cells. The scratch was measured 24 hours later original magnification: 200×. The dotted black line outlines the cell boundary. **B:** Quantification of wound healing was performed in AGS and MGC-803 cells. **C:** The relative percentage of invasion cells of different groups were analyzed. **D:** Representative invasion images of BLACAT2 silenced and Nc cells. Quantifications of cells on the lower surface of the membrane were performed with three randomly chosen fields. Data was presented as means ± SD (*p < 0.05, **p < 0.01, and ***p < 0.001).

To further explore the functional role of BLACAT2 in gastric cancer, we studied the effects of BLACAT2 on gastric cancer cell migration and invasion *in vitro*. Compared to the Nc group, the silencing of BLACAT2 remarkably inhibited AGS and MGC803 cell migration *in vitro* through the wound healing experiment **(Figure 3A).** The relatively wounding closure of different groups was calculated and shown in **Figure 3B**. Moreover, as outlined in **Figure 3D**, the silencing of BLACAT2 decelerated gastric cancer cell invasion *in vitro* by transwell invasion assay. The relative percentage of invasion cells of different groups was analyzed and shown in **Figure 3C**. In short, these data showed that the silencing of BLACAT2 using the shRNA significantly restrained the migration and invasion capacity in the AGS and MGC-803 cells.

### BLACAT2 Sponged miR-193b-5p to function

**Figure 4:**
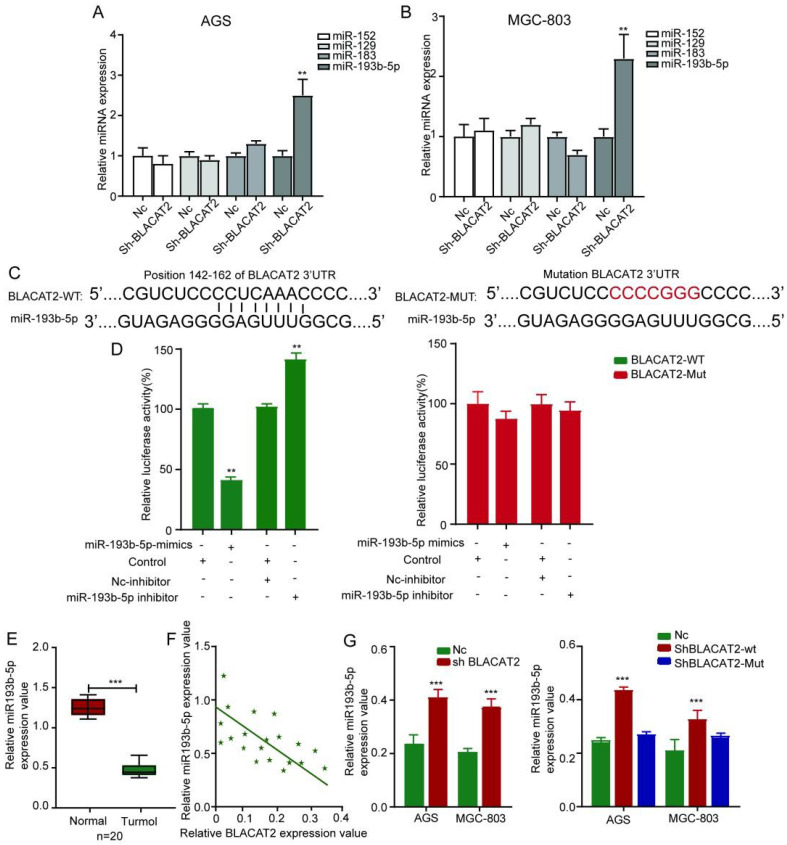
** BLACAT2 sponged miR-193b-5p to function. A-B:** After silencing of BLACAT2 and detecting the expression of all the candidate miRNAs in gastric cancer cells, only miR-193b-5p was remarkably upregulated in response to BLACAT2 silencing in both AGS and MGC-803 cells. **C:** Potential binding site for miR-193b-5p in BLACAT2 was predicted. **D:** Luciferase reporter assay with BLACAT2‐wt or BLACAT2‐mut reporter showed that miR-193b-5p upregulation suppressed the activity of BLACAT2‐wt reporter in AGS cells, miR-193b-5p inhibition enhanced the activity of BLACAT2‐wt reporter in AGS cells, while there was no noticeable alteration for the luciferase activity of BLACAT2‐mut reporter. **E:** Relative expression of miR-193b-5p was tested by qRT‐PCR in 20 matched gastric cancer tissues and normal tissues. **F:** Expression correlation between BLACAT2 and miR-193b-5p in gastric cancer tissues was measured. **G:** BLACAT2 silencing led to increased expression of miR-193b-5p, whereas deletion of the binding site for miR-193b-5p abolished this effect. *p < 0.05, **p < 0.01, and ***p < 0.001. qRT‐PCR: quantitative reverse‐transcription polymerase chain reaction.

Occasionally, it has been confirmed that lncRNAs can function as competitive endogenous RNA (ceRNA) by competitively binding microRNAs. Therefore, we speculated that BLACAT2 could act as a microRNA sponge to function. According to miRDB, TargetScan, and miRWalk databases, we established that several microRNAs such as miR-152, miR-129, miR-183, and miR-193b-5p could bind to BLACAT2. Besides, as illustrated in **Figure 4A-B**, the qRT-PCR experiment validated that the expression of miR-193b-5p significantly increased in both AGS and MGC-803 cell lines upon BLACAT2 depletion, suggesting a regulatory relationship between BLACAT2 and miR-193b-5p. Remarkably, we noticed a potential binding site for miR-193b-5p in BLACAT2, presented in **Figure 4C**. To further confirm the interactive relationship between BLACAT2 and miR-193b-5p, a luciferase reporter assay was steered. Here, the results demonstrated that the miR-193b-5p significantly mimicked transfection, which inhibited the luciferase activity of wt reporter. Notably, the miR-193b-5p inhibitor transfection was also considerably promoted by the luciferase activity of wt reporter in AGS cells. However, as demonstrated in **Figure 4D,** this binding site's mutation abolished these effects. Also, as outlined in **Figure 4E,** the miR-193b-5p expression level was down-regulated in gastric cancer tissues compared to normal tissues, which was opposite to BLACAT2 as previously confirmed.

Additionally, miR-193b-5p in gastric cancer tissues **(Figure 4F)**. More critical, BLACAT2 inhibition promoted the expression of miR-193b-5p in gastric cancer cells, whereas the expression of mutant BLACAT2 (deletion of the potential binding site for miR-193b-5p) did not affect miR-193b-5p expression **(Figure 4G)**. Collectively, BLACAT2 interacted with miR-193b-5p to inhibit its availability in gastric cancer.

### MiR-193b-5p inhibition rescued the proliferation and apoptosis effects affected by BLACAT2 silencing

**Figure 5:**
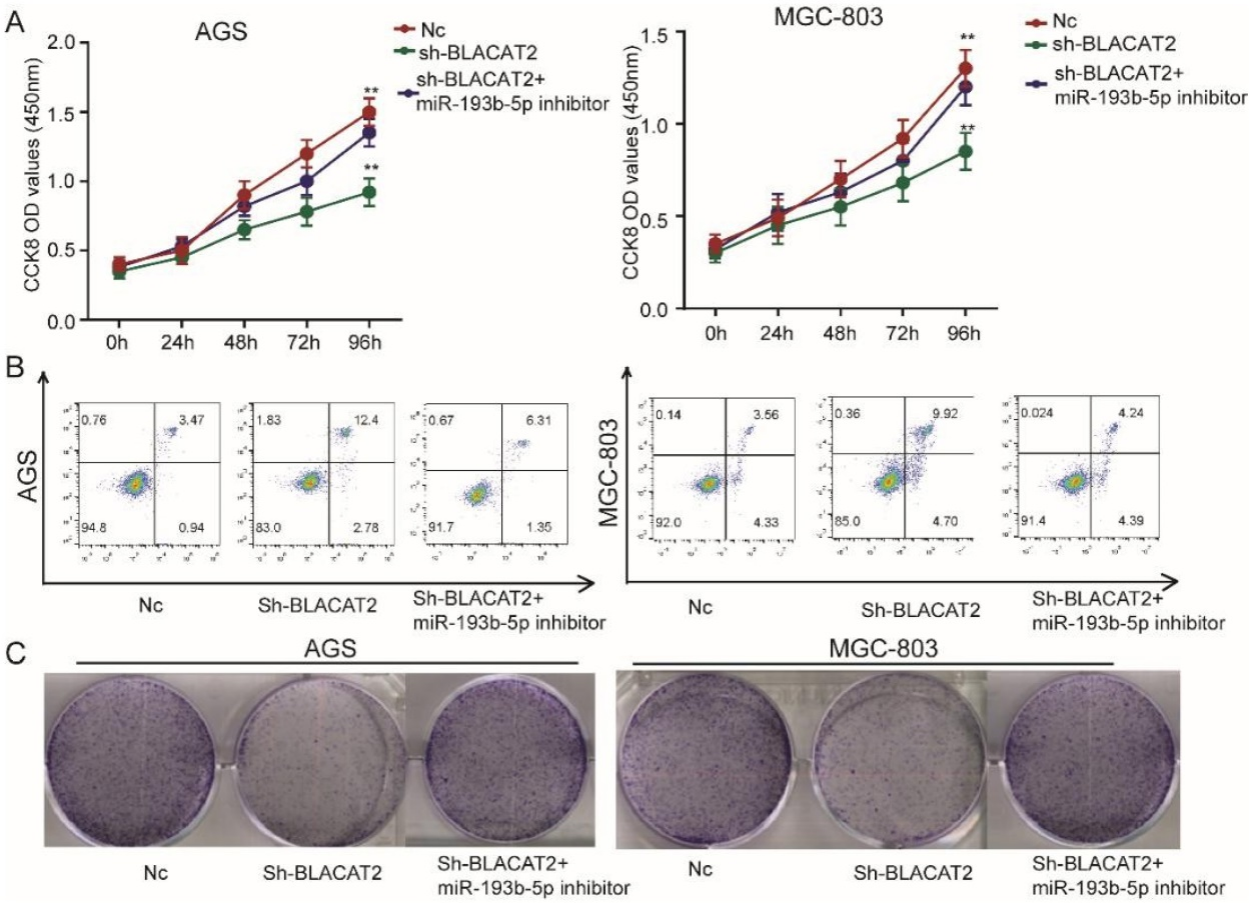
** Inhibition of miR-193b-5p abolished the effects of BLACAT2 silencing in gastric cancer cells. A:** CCK8 assay demonstrated that miR‐193b-5p inhibitor transfection restored the proliferation of AGS and MGC-803 cells transfected with sh-BLACAT2. **B:** Flow cytometry detection showed that miR‐193b-5p inhibition restored the promotion effects of apoptosis of AGS and MGC-803 cells transfected with sh-BLACAT2. **C:** Plate colony formation assay revealed that miR-193b-5p inhibition rescued AGS's proliferation capacity and MGC-803 cells affected by BLACAT2 silencing. Data was presented as means ± SD (*p < 0.05, **p < 0.01, and ***p < 0.001).

To date, the exact biological function of miR-193b-5p has not been explored in gastric cancer. Therefore, in this study, we transduced the miR-193b-5p inhibitors to test whether BLACAT2 exerts its functional roles through miR-193b-5p. First, as outlined in **Figure 5A**, we discovered that miR-193b-5p inhibition significantly rescued the proliferation capacity of BLACAT2-silenced AGS and MGC-803 cells through the CCK8 assays. Secondly, flow cytometry experiments revealed that the miR-193b-5p inhibition eliminated the promoting effects of apoptosis induced through the BLACAT2-knockdown in both the AGS and MGC-803 cells **(Figure 5B)**; the statistical results are presented in **Supplementary Figure 2A-B**. Thirdly, plate colony formation assays showed that the down-regulation of miR-193b-5p enhanced increased proliferation capacity of gastric cancer cell lines *in vitro* **(Figure 5C)**; the statistical analysis results are presented in **Supplementary Figure 2C-D.** In summary, miR-193b-5p acted as a tumor suppressor and was negatively regulated by BLACAT2 in gastric cancer.

### METTL3 was a direct target of miR-193b-5p

**Figure 6:**
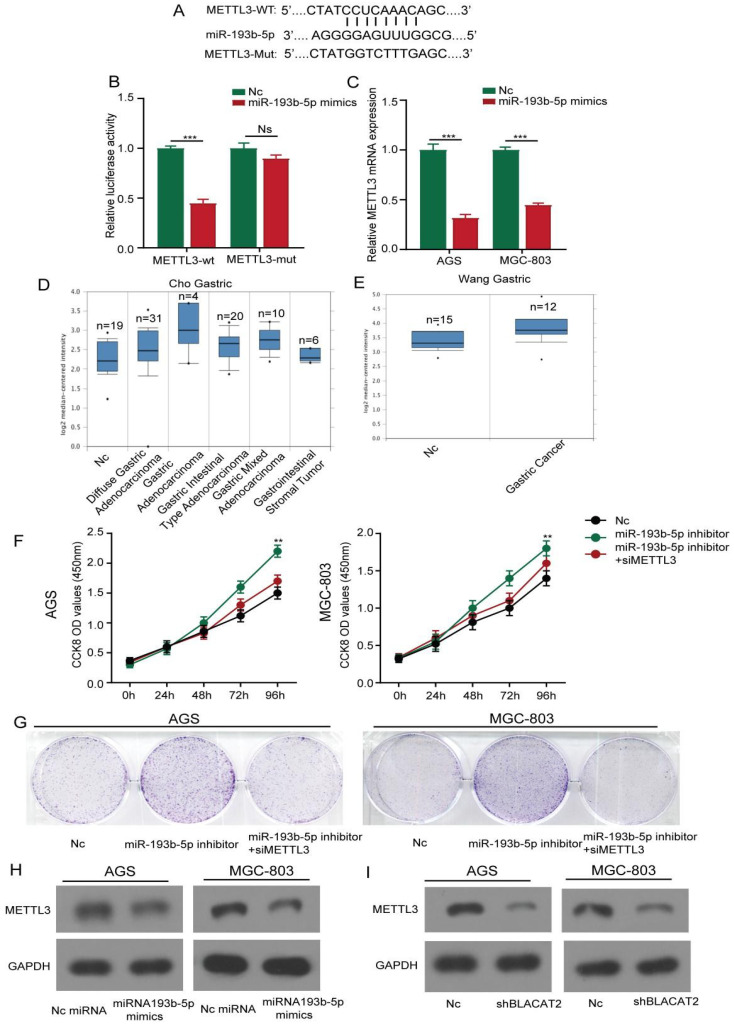
** METTL3 was a direct target of miR-193b-5p and positively regulated by BLACAT2. A:** The predicted potential binding sites of miR-193b-5p with the 3′‐UTR of METTL3. The mutated version of the METTL3 3′‐UTR was also given. **B:** Luciferase reporter assay showed that miR‐193b-5p mimic suppressed the activity of METTL3 3′‐UTR‐wt reporter in AGS cells. **C:** MiR-193b-5p overexpression significantly inhibited the expression of METTL3 in gastric cancer cells at mRNA levels through qRT‐PCR. **D-E:** Expression intensity of METTL3 was analyzed in gastric cancer tissues and normal control tissues according to the oncomine dataset. **F-G:** CCK8 and plate colony formation experiments demonstrated that METTL3 knockdown partly abolished the promotion effects of the proliferation of AGS and MGC-803 cells transfected with miR‐193b-5p inhibitor. **H:** Flow cytometry experiment proved that METTL3 knockdown partly weakened the inhibitory effects of apoptosis of AGS and MGC-803 cells transfected with miR‐193b-5p inhibitor. **I:** METTL3 expression was repressed by miR-193b-5p mimics in AGS and MGC803 cells at the protein level by western-blot analysis. **J:** Relative expression of METTL3 was suppressed by BLACAT2 silencing confirmed by western-blot analysis. Data was presented as means ± SD (*p < 0.05, **p < 0.01, and ***p < 0.001, Ns : Statistically no sense).

As illustrated in **Figure 6A**, we further predicted the potential target genes of miR-193b-5p through bioinformatics analysis and identified that METTL3 3′‐UTR contains a possible binding site miR-193b-5p. Subsequently, we used the luciferase reporter experiment to test whether miR-193b-5p directly bound to METTL3. As shown in **Figure 6B**, this study discovered that miR-193b-5p mimicked transfection and significantly repressed the luciferase intensity in AGS cells transfected with METTL3‐wt reporter, rather than METTL3‐mut reporter. Next, we performed qRT‐PCR analysis and established that the overexpression of miR-193b-5p significantly inhibited the expression of METTL3 in gastric cancer cells at mRNA levels **(Figure 6C).** To further assess the expression status of METTL3 in human gastric cancer tissues, we first analyzed the mRNA expression levels of METTL3 in oncomine datasets. As shown in **Figure 6D-E**, the results revealed that the METTL3 expression level was elevated dramatically in various gastric cancer types compared with normal tissues.

Consequently, we investigated the effects of miR-193b-5p on the proliferation of gastric cancer cell lines. Here, the results showed that inhibition of miR-193b-5p promoted AGS cell proliferation activity, MGC803 cell lines through the CCK8, and plate colony formation assays. In contrast, the METTL3 inhibition partly abolished the effects **(Figure 6F-G)**; the statistical results are presented in **Supplementary Figure 3A-B.** Our results were consistent with previous results because apoptosis flow cytometry experiments demonstrated that inhibition of miR-193b-5p suppressed cell apoptosis, whereas inhibition of METTL3 partly reversed the effects **(Supplementary Figure 3C)**. Moreover, we performed western blot analysis and established that the overexpression of miR-193b-5p significantly inhibited the expression of METTL3 in gastric cancer cells at different protein levels **(Figure 6H).** Besides, as illustrated in **Figure 6I,** we further confirmed that the silencing of BLACAT2 genes suppressed METTL3 expression in AGS and MGC803 cells. In summary, our data demonstrated that METTL3 was a direct target of miR-193b-5p and positively regulated by BLACAT2.

### Restoration of METTL3 expression abolished the effects of BLACAT2 silencing

**Figure 7:**
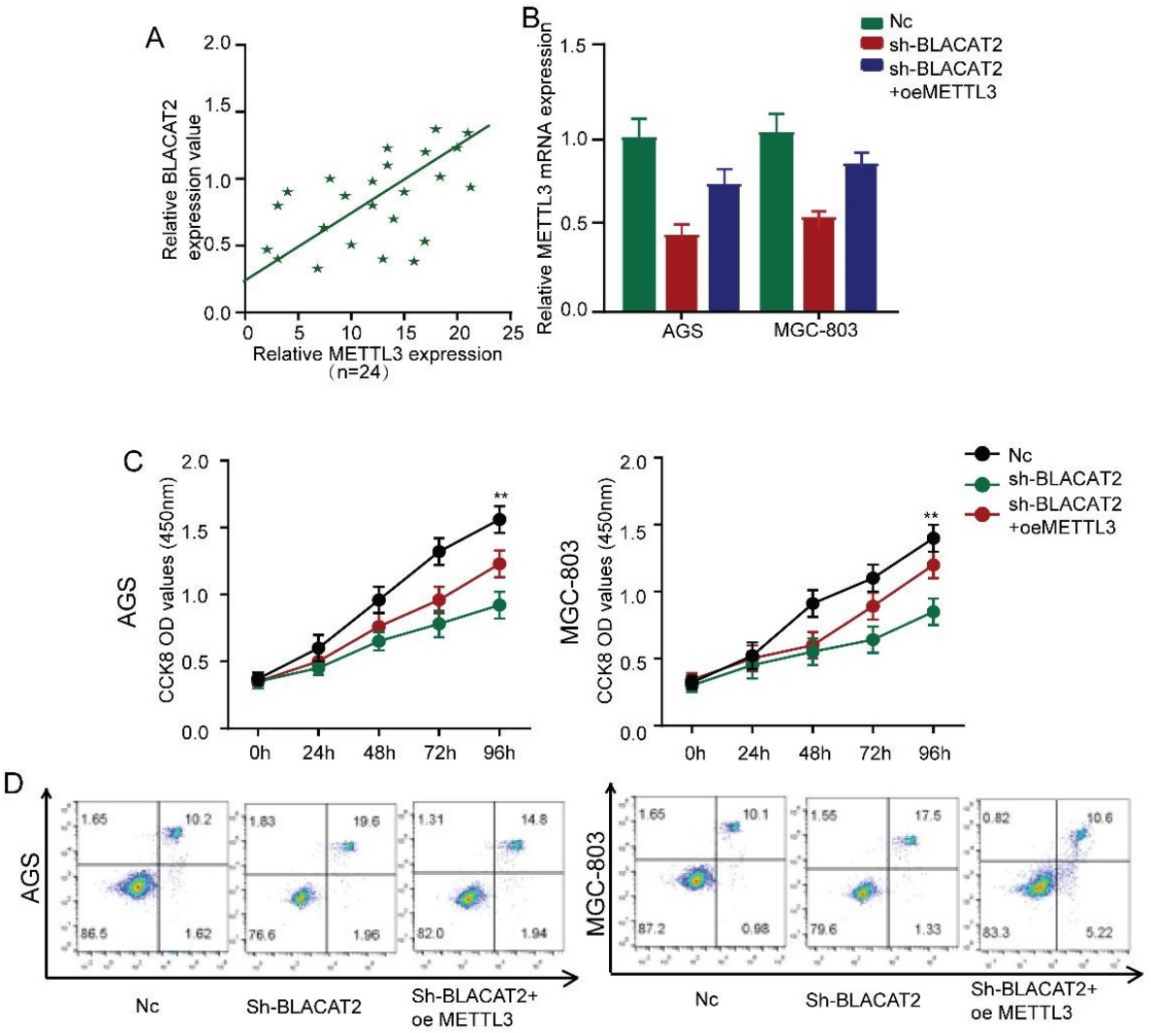
** Restoration of METTL3 expression abolished the effects of BLACAT2 silencing. A:** Expression correlation between METTL3 and BLACAT2 in gastric cancer tissues was determined (n=24). **B:** Relative expression of METTL3 was analyzed by qRT‐PCR in AGS and MGC-803 cells transfected with indicated plasmids. **C:** CCK8 assay demonstrated that METTL3 overexpression restored the proliferation of AGS and MGC-803 cells transfected with shBLACAT2. **D:** Flow cytometry detection showed that METTL3 overexpression partly restored the promotion effects of apoptosis of AGS and MGC-803 cells transfected with shBLACAT2. Data was presented as means ± SD (*p < 0.05, **p < 0.01, and ***p < 0.001. oe: overexpression).

The above experimental results compelled us to test whether BLACAT2 regulates the expression of METTL3 by inhibiting miR-193b-5p. Interestingly, as demonstrated in **Figure 7A**, we noticed a positive expression correlation between BLACAT2 and METTL3 in gastric cancer tissues.

Besides, **Figure 7B** explains the silencing of BLACAT2, which decreased the expression of METTL3 in gastric cancer cells. However, these effects were reversed through the overexpression of METTL3. Subsequently, we restored the METTL3 expression in BLACAT2‐silenced cells to perform a rescue experiment. Here, as illustrated in **Figure 7C**, we established that the overexpression of METTL3 substantially rescued the proliferation capacity of BLACAT2- silenced AGS and MGC-803 cells through CCK8 assays; the statistical outcomes are shown in **Supplementary Figure 4A-B.**

Flow cytometry experiments revealed that METTL3 restoration partly eliminated apoptosis's promoting effects, induced due to BLACAT2-knockdown in both AGS and MGC-803 cells **(Figure 7D).** Plate colony formation assay showed that the METTL3 restoration partly reversed the suppressive effects of proliferation induced by BLACAT2-knockdown in both AGS and MGC-803 cells **(Supplementary Figure 4C-D).** In a nutshell, these data proved that BLACAT2 promotes gastric cancer progression by upregulating the expression of METTL3, which inhibits miR-193b-5p.

### METTL3 expression was downregulated in BLACAT2 silenced xenograft tumor-bearing tissues

To further verify whether BLACAT2 regulated METTL3 in tumor-bearing tissues, we determined the expression of METTL3 in the tumors (from **Figure 2E**) using immunohistochemical (IHC) staining. The results showed that METTL3 expression levels in AGS/shBLACAT2 derived tumors were remarkedly lower than Nc derived tumors **(Supplementary Figure 5A).** Moreover, tumor regression was accompanied by significantly decreased expression levels of PCNA, a key index of proliferation **(Supplementary Figure 5B).**

## Discussion

Different studies on non-coding RNAs (lncRNAs) have recently been proved to participate in regulating the initiation and progression of human tumors by a growing number of studies in recent years 14, 15. The abnormal lncRNA expression was relative to developing different cancers, including gastric cancer 16. Our study used a gene-wide expression profiling chip analysis, which identified differentially expressed lncRNA-BLACAT2 in gastric cancer for the first time. Also, we established that BLACAT2 was upregulated in tumor samples compared with the adjacent normal tissues.

More experiments showed that silencing of BLACAT2 inhibited gastric cancer cell proliferation, migration, and invasion both in *in vitro* and *in vivo* setups as the imbalance of cell apoptosis and proliferation is an important event in cancer progression 17. Notably, the BLACAT2 knockdown enhanced the apoptosis of gastric cancer cells. Previously, the regulatory mechanism of lncRNAs in transcriptomes, like chromatin modification, post-transcriptional, and transcriptional processing, has been fully confirmed 18, 19. In this study, we observed that BLACAT2 directly interacts with miR-193b-5p. It functions as an oncogene in gastric cancer through sponging miR-193b-5p, which exerts its effects by regulating the expression of the downstream target gene METTL3. Our findings demonstrated that BLACAT2 plays a critical oncogenic role during gastric cancer progression.

BLACAT2, also known as lncRNA-00958, has been identified to play oncogenic roles and participate in several tumor occurrence processes. Recently, research demonstrated an elevated link between BLACAT2 expression with metastasis and unfavourable prognosis in gastric cancer 20. Consistent with previous findings, in our study, we measured the expression patterns of BLACAT2 in gastric cancer. Through gene-wide expression profiling chip analysis and qRT‐PCR analysis, it was observed that BLACAT2 was upregulated in gastric cancer. Subsequently, we discovered that lymph node metastasis or vascular invasion patients displayed a relatively higher BLACAT2 expression. Notably, the amount of BALCAT2 expression increased with the T stage increase in gastric cancer tissues, suggesting that BLACAT2 could be a prognostic indicator. Furthermore, the silencing of BLACAT2 affected the biological functions such as proliferation and apoptosis of gastric cancer cells, signifying BALCAT2 could be a therapeutic target for gastric cancer treatment.

In the ceRNA network, lncRNAs protected downstream genes from the mRNA repression or degradation in translation caused by miRNAs to upregulate gene expression 21. MiRNAs belong to small non-coding RNAs with a length of about 20 to 24 nucleotides that function in the progression and development of different cancers 22. Several studies have suggested that lncRNAs, including BLACAT2, regulates cancer progression by sponging miRNAs. For instance, BLACAT2 has been shown to facilitate cervical cancer cell proliferation and metastasis by sponging miR-625-5p, which upregulates the expression of LRRC8E 23. Similarly, it was verified that the silencing of BLACAT2 prevents tumor initiation of pancreatic cancer by acting as a sponge of microRNA-330-5p to down-regulate PAX8 24.

Our study established that BLACAT2 could interact with miR-193b-5p and inhibit the expression of miR-193b-5p. Consequently, the luciferase reporter assay confirmed the interactive relationship between them. Although previous studies have validated that miR-193b-5p acts as an endogenous tumor suppressor in several malignancies such as breast cancer 25, and acute myeloid leukemia 26, the precise role of miR-193b-5p in gastric cancer remains uncertain. Here, we established that in gastric cancer tissues, there was a remarkable down-regulation of miR-193b-5p. Also, BLACAT2 negatively regulated the expression of MiR-193b-5p. On the other hand, rescue experiments illustrated that miR-193b-5p inhibition could reverse the promoting function of BLACAT2 silencing on cell proliferation, metastasis, and apoptosis, which indicates that BLACAT2 promoted gastric cancer progression by serving as a miR-193b-5p sponge.

For the first time, this study established that miR-193b-5p directly targets N6-Adenosine-Methyltransferase 70 KDa Subunit (METTL3) in gastric cancer. METTL3, the critical component of the N6-methyltransferase complex, has been essential for tumor progression in various cancers. Previous research has suggested that METTL3 is an oncogene in different cancers, like colorectal carcinoma, bladder cancer, and pancreatic cancer 27-29. Besides, other studies have shown that METTL3 plays an essential role in gastrointestinal tumors. Other studies have shown that METTL3 is closely associated with the processes involved in the progression of gastrointestinal cancer, including tumor proliferation, apoptosis, metastasis, angiogenesis, chemo/radiotherapy resistance, glycolipid metabolism, and cancer stem cell (CSC) maintenance 30, 31.

As for gastric cancer, studies confirmed that METTL3 is a vital tumor promoter. The METTL3-mediated N6-methyladenosine modification is vital for epithelial-mesenchymal transition and metastasis of gastric cancer 32. Another significant research demonstrated that METTL3 promotes gastric cancer progression and has prognostic significance 33. Moreover, upstream regulatory molecules of METTL3 in gastric cancer have also been discovered and confirmed in lots of researches in recent years. For instance, it was proved that miR-4429 prevents gastric cancer progression by targeting METTL3 34. P300-mediated H3K27 acetylation activation present in the promoter region of METTL3 induced the transcription of METTL3, thus promoting tumor angiogenesis and glycolysis in gastric cancer 35.

As METTL3 was the primary regulator involved in the abundant m6A RNA modification, the relations between lncRNAs and m6A modification have also emerged as research hotspots in the initiation and progression of gastric cancer. A recent study demonstrated that impaired autophagic degradation of lncRNA ARHGAP5-AS1 in chemoresistant cancer cells promoted chemoresistance by activating the transcription of ARHGAP5 in the nucleus and stimulate m6A modification of ARHGAP5 mRNA to stabilize ARHGAP5 mRNA in the cytoplasm by recruiting METTL3^35^, ^36^. Consistently, our study also verified the oncogenic role of METTL3 in gastric cancer. Here, we established that the BLACAT2 induced suppression of miR-193b-5p and upregulated the METTL3 expression. Additionally, rescue assays demonstrated that BLACAT2 exerts oncogenic roles, which rely on the expression of METTL3 in gastric cancer.

In conclusion, our study demonstrates that lncRNA BLACAT2 is significantly upregulated in gastric cancer tissues. Besides, our subsequent experiments have suggested that BLACAT2 promotes gastric cancer proliferation, migration, and invasion but inhibits apoptosis by regulating the miR-193b-5p/ METTL3 axis. Therefore, this study has revealed that BLACAT2 can act as an oncogene in gastric cancer. However, there is a need for further studies to clarify the molecular mechanism that may facilitate the development of a new candidate for targeted therapy of gastric cancer.

## Supplementary Material

Supplementary figures.
