# Supplementary material for: Longnon-coding RNA BLACAT2 promotes gastric cancer progression via the miR-193b-5p/METTL3 pathway
Source: J Cancer. 2021 Apr 2;12(11):3209–21. doi: 10.7150/jca.50403 (PMC8100803; doi:10.7150/jca.50403)
Supplement: Supplementary file 1 — Supplementary figures. [file jcav12p3209s1.pdf]

Supplementary Figure 1.

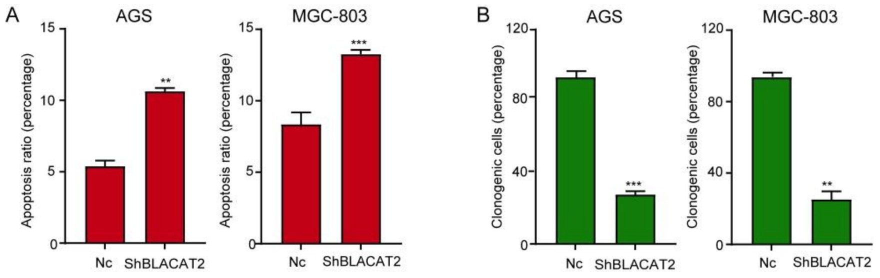

A: The statistical results of Figure 2C. **B:** The statistical results of Figure 2D.

(*p < 0.05, **p < 0.01and ***p < 0.001).

Supplementary Figure 2.

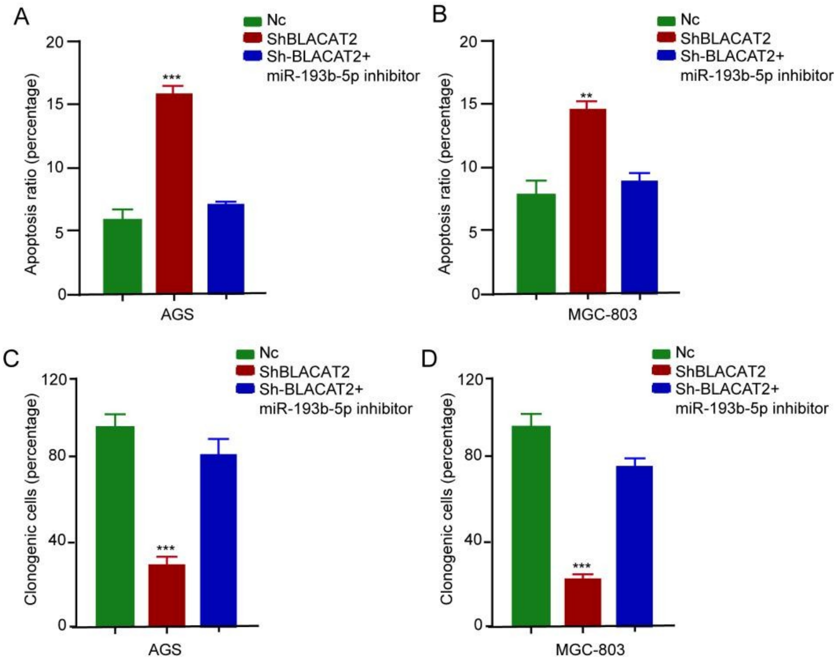

A-B: The statistical results of Figure 5B. **C-D:** The statistical results of Figure 5C. (*p < 0.05, **p < 0.01and ***p < 0.001).

Supplementary Figure 3.

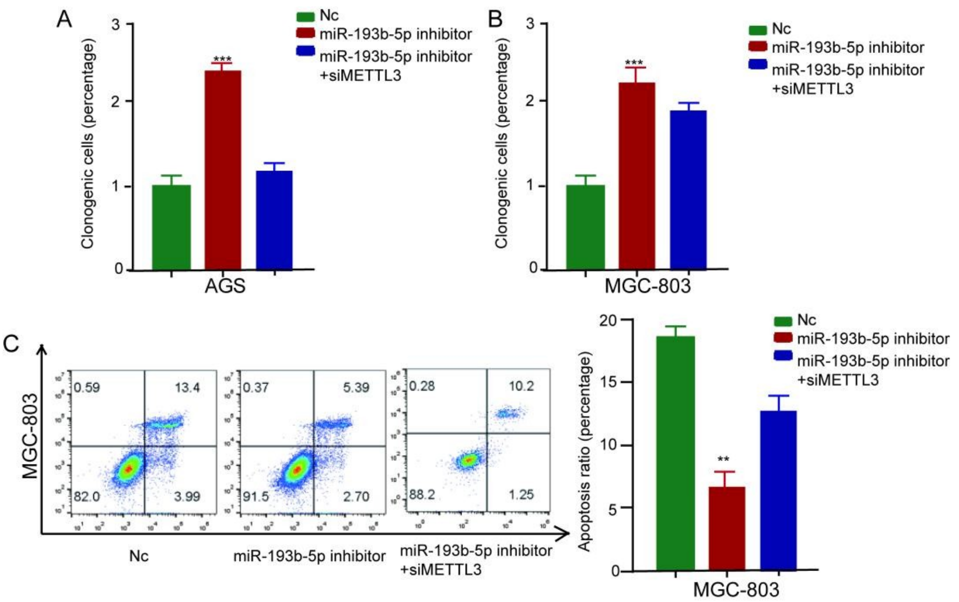

A-B: The statistical results of Figure 6G. **C:** Apoptosis flow cytometry experiments demonstrated that inhibition of miR-193b-5p suppressed cell apoptosis while inhibiting METTL3 partly reversed the effects. the statistical results were shown on the right. (*$p < 0.05$, **$p < 0.01$and***$p < 0.001$).

Supplementary Figure 4.

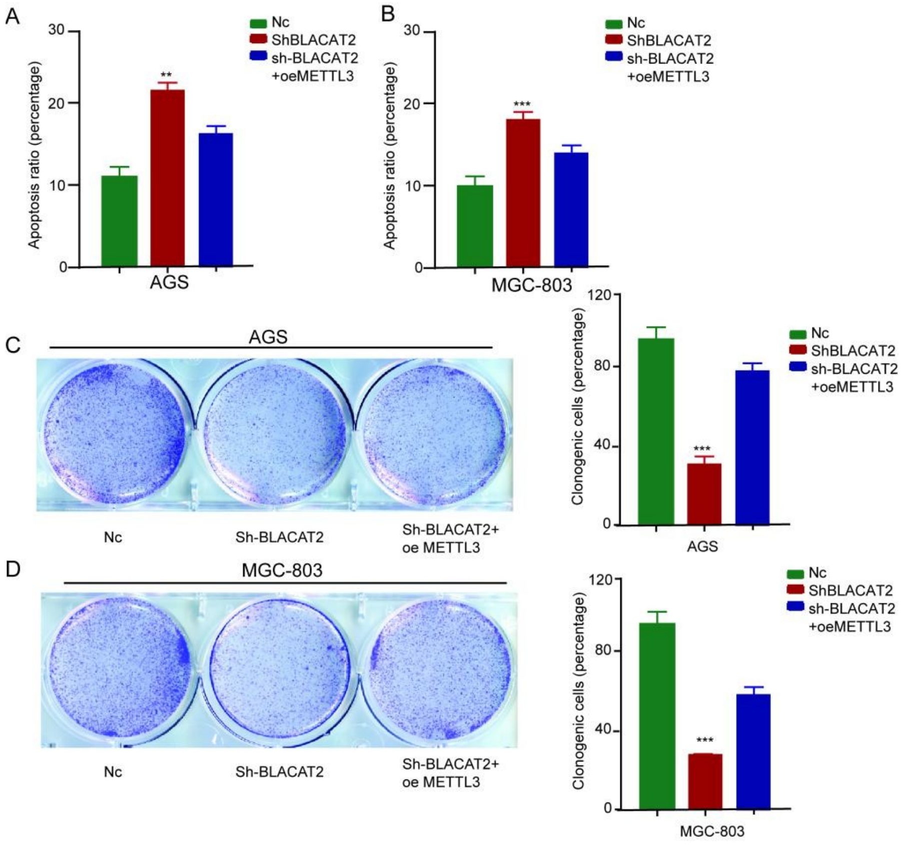

A-B: The statistical results of Figure 7D. **C-D:** Plate colony formation assay showed that METTL3 restoration partly reverses the suppressive effects of proliferation induced by BLACAT2-knockdown in both AGS and MGC-803 cells. the statistical results were shown on the right. (*p < 0.05, **p < 0.01and ***p < 0.001).

Supplementary Figure 5. METTL3 expression was downregulated in BLACAT2 silencing xenograft tumor-bearing tissues.

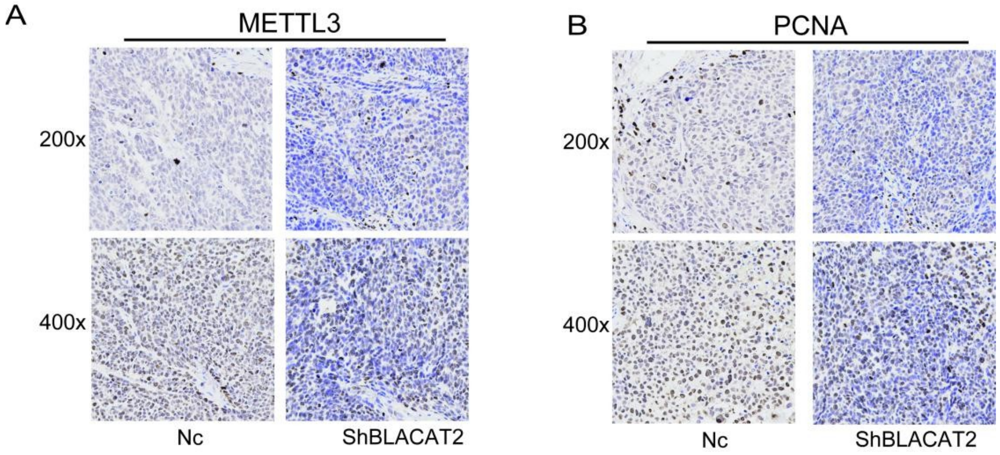

A: Decreased expression of METTL3 in tissues from AGS/shBLACAT2 was detected than Nc by IHC staining. **B:** Compared with Nc mice, PCNA expression was also down-regulated in BLACAT2 silencing tissues.
